# Insomnia Symptoms, Mental Health Diagnosis, Mental Health Care Utilization, and Perceived Barriers in U.S. Males and Females

**DOI:** 10.3390/jcm14092989

**Published:** 2025-04-25

**Authors:** Wendemi Sawadogo, Anuli Njoku, Joy Jegede

**Affiliations:** 1 Department of Public Health, College of Health and Human Services, Southern Connecticut State University, New Haven, CT 06515, USA; njokua3@southernct.edu; 2 Department of Social Work, College of Health and Human Services, Southern Connecticut State University, New Haven, CT 06515, USA; jegedej1@southernct.edu

**Keywords:** insomnia symptoms, mental health, mental health care, anxiety, depression, females, males, health disparities

## Abstract

**Objective:** We aim to determine the association between insomnia symptoms and mental health in females and males and compare mental health care utilization and perceived barriers between females and males with insomnia symptoms. **Methods**: This is a cross-sectional study using the National Health Interview Survey. Insomnia symptoms included self-reported “trouble falling asleep”, ‘trouble staying asleep”, and “waking up feeling not well rested”. Mental health included self-reported anxiety and depression. Multivariable logistic regression was used to assess the association between insomnia symptoms and mental health in females and males. **Results**: A total of 26,691 adults were included. The mean age was 48.2 years; 51.4% were females, and 48.6% were males. Insomnia symptoms were associated with anxiety and depression for both females and males. These associations were stronger in younger adults (<50 years) than older adults (≥50 years). Females with insomnia symptoms were more likely to receive mental health care (OR = 1.7; 95% CI = 1.53, 1.87) but also to delay mental health care because of its cost (OR = 1.96; 95% CI: 1.67, 2.30) or needed mental health care but did not get it because of the cost (OR = 2.14; 95% CI: 1.82, 2.50) than their males counterpart. **Conclusions**: Insomnia symptoms were associated with mental health in females and males, being stronger in younger adults than older adults, with gender differences in mental health care utilization and financial barriers to mental health care. Holistic approaches involving prevention and better access to mental health care are warranted.

## 1. Introduction

The growing prevalence of mental health disorders that affect individuals’ physical and social well-being makes mental health a public health issue in the United States. It is estimated that more than one in five U.S. adults live with a mental illness (59.3 million in 2022; 23.1% of the U.S. adult population) [1]. Anxiety and depression are the most common mental health disorders in the U.S. During 2022, about one in five adults age 18 and older experienced any symptoms of anxiety (18.2%) or depression (21.4%) in the past two weeks [2]. Despite the burden of mental health, access to mental health care remains a challenge, with only 47% of adults with mental illness receiving treatment [1]. Cost is the most common barrier, with over 60% of adults citing it as a reason for not seeking care. A lack of insurance, stigma, and lack of access to health care providers are also significant barriers [3,4].

Insomnia is an experience associated with persistent difficulty initiating sleep, difficulty maintaining sleep, or waking up too early, despite adequate opportunity to sleep [5,6]. About 10% of the adult population experiences chronic insomnia, and 30% experience short-term insomnia [7]. Insomnia and sleep disorders in general constitute significant public health challenges, resulting in high health care costs while reducing work productivity.

The relationship between mental health and insomnia is complex; mental health is commonly associated with insomnia and vice versa [8]. The human body is designed to require a regular pattern for sleep, a necessary biological function to ensure healthy cognition, emotional, mental, and social well-being [5]. Insomnia disrupts this biological function and may stand as an isolated disorder, a co-occurring condition with a mental disorder, or a consequence of a mental disorder [9]. Previous longitudinal studies have reported a correlation between insomnia and depression [10,11]. A meta-analysis of longitudinal studies reported that insomnia was associated with a 3-fold increased risk of presenting anxiety disorder (pooled OR: 3.23; CI 1.52–6.85) [12]. In another meta-analysis of longitudinal studies, insomnia symptoms were independently associated with depression (pooled RR: 2.27; 95% CI: 1.89–2.71) [13]. The experience of insomnia could trigger depressive symptoms where they were previously absent.

While sex differences in insomnia patterns, on one hand, and mental health disorders, on the other hand, have been separately reported, few studies have investigated whether the association between insomnia and mental health differs by sex [14,15,16,17]. Differences between females and males were reported in the prevalence and incidence of sleep disorders and mental health. Females are more likely to report insomnia symptoms, receive a diagnosis of insomnia, and are more frequently diagnosed with mental health disorders such as anxiety and depression than males [14,15,16,17]. Suggested reasons include biological, hormonal, psychosocial, and socioeconomic factors. For example, sex hormones exert a significant influence on circadian rhythms and sleep, with estrogen, testosterone, and progesterone all playing a role in regulating sleep–wake cycles, mood, and other physiological processes [18]. It is, however, unclear if the association between insomnia symptoms and mental health differs by sex. We hypothesized that those differences would be reflected in the association between insomnia symptoms and mental health and, thus, require separate investigation. Targeted interventions may contribute to addressing the complex yet increasing mental health burden. Furthermore, males and females with insomnia symptoms may face different barriers to accessing mental health care. Using cross-sectional data representative of the U.S. adult population, this study aimed to address four objectives. First, we examined the association between insomnia symptoms and mental health in females and males. Second, we explored the moderating role of race and ethnicity, place of residence, and age. Third, we compared mental health care utilization and perceived barriers between females and males with insomnia symptoms. Fourth, we investigated potential moderation and mediation by place of residence and insurance coverage.

## 2. Materials and Methods

### 2.1. Data Source and Study Design

This cross-sectional study used publicly available and de-identified data from the 2022 National Health Interview Survey (NHIS) [19]. NHIS is an annual household survey representative of the civilian noninstitutionalized population in the United States conducted by the National Center for Health Statistics (NCHS) of the Centers for Disease Control and Prevention (CDC). To that end, the NHIS uses a complex sample design involving stratification and clustering. The main objective of the NHIS is to monitor the health of the United States population through the collection and analysis of data on a broad range of health topics.

The survey is conducted continuously throughout the year. Interviews are conducted in respondents’ homes and/or over the telephone. Informed consent is obtained from the participants. Information about the Sample Adult is self-reported, unless physically or mentally unable to do so, then a knowledgeable proxy answers for the Sample Adult. This study used data from the Sample Adults (age ≥ 18 years). In 2022, there were 27,651 Sample Adult interviews. The 2022 survey included, in the Sample Adult, items related to sleep and mental health assessment for depression and anxiety. As these items were rotating and not included in all surveys, the 2022 survey was appropriate for this research question.

### 2.2. Insomnia Symptoms

Participants were asked “during the past 30 days, how often did you wake up feeling well-rested?”; “how often did you have trouble falling asleep?”; and “how often did you have trouble staying asleep?”. The response options were “never”, “some days”, “most days”, “every day”, “refused”, and “don’t know”. Those who endorsed “never” received a score of 0, “some days” a score of 1, “most days” a score of 2, and “every day” a score of 3. Missing values were assigned to “refused” and “don’t know”. Reverse coding was used for the question related to “wake up feeling well-rested”. The total insomnia score ranges from 0 to 9 with higher scores indicating severe insomnia symptoms.

### 2.3. Mental Health

Dichotomous variables were created for mental health diagnosis based on the answers to questions related to anxiety (Have you ever been told by a doctor or other health professional that you had any type of anxiety disorder?) and depression (Have you ever been told by a doctor or other health professional that you had any type of depression?). Similarly, mental health care utilization and perceived barriers were based on the following questions: “during the past 12 months, did you receive counseling or therapy from a mental health professional such as a psychiatrist, psychologist, psychiatric nurse, or clinical social worker?”, “have you delayed getting counseling or therapy from a mental health professional because of the cost?”, and “was there any time when you needed counseling or therapy from a mental health professional but did not get it because of the cost?”.

### 2.4. Covariates

Based on the literature review and the available data, the covariates included age, sex (male or female), race and ethnicity (Hispanic, Non-Hispanic Asian, Non-Hispanic Black, Non-Hispanic White, and Other), education level (less than high school, high school graduate, some college, associate, bachelor, and graduate degree), marital status (married, in couple, or neither), ratio of family income to poverty threshold, smoking (current, former, or never), alcohol consumption (current, former, or abstainer), physical activity (inactive, insufficiently active, or sufficiently active), body mass index (underweight, normal weight, overweight, or obese), diabetes (yes or no), hypertension (yes or no), heart disease (yes or no), stroke (yes or no), cancer (yes or no), and sleeping pill utilization.

### 2.5. Statistical Analysis

Descriptive statistics were used to summarize the participants’ characteristics by insomnia symptom scores. Correlations between categorical variables were assessed using the phi coefficient. Multicollinearity was tested using the variance inflation factor. Logistic regressions were developed to assess the association between insomnia symptoms and anxiety, as well as the association between insomnia symptoms and depression in males and females in the study population. In addition, logistic regression models were developed to compare mental health care utilization and perceived barriers in males and females with insomnia symptoms. This analysis was restricted to those with insomnia symptoms, as they are the ones most likely to seek health care. Moderation was assessed by adding a multiplicative interaction term in the model. Mediation was assessed using the counterfactual framework, which decomposed the total effect into natural indirect and direct effects [20]. The mediated proportion was computed as the natural indirect effect divided by the total effect, and the 95% CI was estimated by repeating 1000 bootstrapped samples. The models were adjusted for the covariates listed above. The model fit was checked using the Hosmer–Lemeshow goodness-of-fit test. Further analysis was conducted for individual insomnia symptoms by categorizing each insomnia symptom into two groups. All statistical analyses were performed in SAS^®^ 9.4 software with an alpha level set at 0.05 (SAS Institute Inc., Cary, NC, USA).

## 3. Results

### 3.1. Description of Study Participants

A total of 26,691 adults who responded to the insomnia questions were included in the present analysis. The mean age was 48.2 years (95% CI: 47.9, 48.5), 51.4% were females and 48.6% were males (Table 1). Insomnia symptom scores were higher in females, Non-Hispanic Whites, those with a low income, smokers, those who were physically inactive, those with a high BMI, and with any comorbidity such as diabetes, hypertension, heart disease, stroke, and cancer.

### 3.2. Association Between Insomnia Symptom Scores and Mental Health

**Figure 1:**
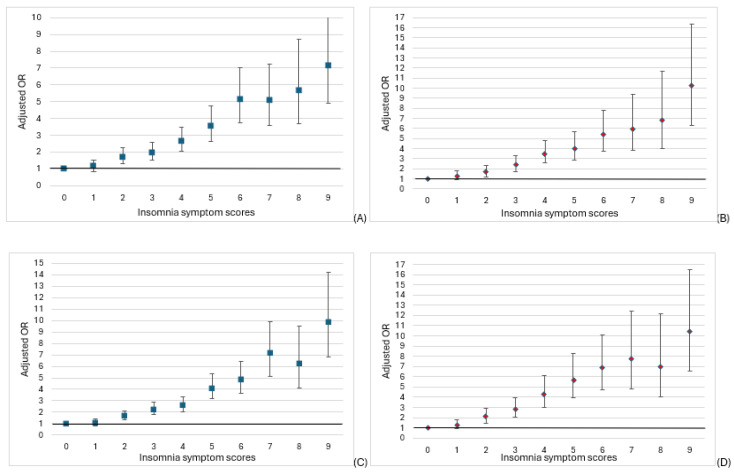
Association between insomnia symptom scores and (**A**) anxiety in females, (**B**) anxiety in males, (**C**) depression in females, and (**D**) depression in males.

After adjusting for all the covariates, the association between insomnia symptoms and mental health outcomes remained significant (Table 2). Every one-unit increase in insomnia symptom scores was associated with 34% increased odds of anxiety for females (OR = 1.34; 95% CI = 1.31, 1.38) and 38% for males (OR = 1.38; 94% CI: 1.33, 1.42). Similarly, insomnia symptoms were associated with depression for both females (OR = 1.38; 95% CI: 1.34, 1.41) and males (OR = 1.40; 95% CI: 1.36, 1.44). The association was slightly higher for males than for females. A clear dose-response was observed between insomnia symptoms and mental health; as insomnia symptom scores increased, the odds of anxiety and depression also increased in both females and males (Figure 1).

The association between insomnia symptoms and mental health outcomes was different by age in both males and females (Table 2). The association was stronger in younger (<50 years) than older adults (≥50 years). For example, females aged <50 years had substantially higher odds of anxiety (OR: 1.34; 95%: 1.29, 1.40) compared to females aged ≥50 years (OR: 1.20; 95% CI: 1.29, 1.40). This pattern was consistent for anxiety and depression in both males and females. However, there was no difference by race and ethnicity, or place of residence (metropolitan or nonmetropolitan). In the individual insomnia symptoms analysis, all three symptoms (difficulty initiating sleep, difficulty maintaining sleep, and non-restorative sleep) were associated with anxiety and depression.

### 3.3. Mental Health Care Utilization and Barriers in Those with Insomnia Symptoms

After adjusting for all covariates, there was a slight increase in the odds ratios compared to the unadjusted model (Table 3), with females with insomnia symptoms having almost 2 times the odds of receiving mental health care in the past year (OR = 1.7; 95% CI = 1.53, 1.87), delaying mental health care because of its cost (OR = 1.96; 95% CI: 1.67, 2.30), and needing mental health care but not getting it because of the cost (OR = 2.14; 95% CI: 1.82, 2.50) than their males counterpart.

Insurance coverage and place of residence (metropolitan or nonmetropolitan) were further assessed for moderation and mediation. While there was no evidence of moderation, a significant albeit minimal mediation by insurance coverage and place of residence was observed (Table 4).

## 4. Discussion

In this study, we found that insomnia symptoms were associated with anxiety and depression in both females and males. The association was slightly stronger in males than in females and more substantial in younger adults than older adults. Females with insomnia symptoms were more likely to have received mental health care in the past year and more likely to experience financial barriers to mental health care than males. These differences in mental health utilization and perceived barriers were partially mediated by insurance coverage and place of residence.

The findings were consistent with previous studies that linked insomnia symptoms to anxiety and depression [10,11,12,13]. Similar to our study, Jaussent et al. utilized self-reported insomnia symptoms, but their study was longitudinal and included older adults from France [10]. Neckelmann et al. included a population from Norway with an age distribution comparable to our sample and also used self-reported insomnia symptoms [11]. Both studies reported that insomnia symptoms were associated with anxiety and depression. Our findings contribute to the existing literature by supporting a dose–response relationship in which the likelihood of depression and anxiety increases with higher levels of insomnia. Although the sex difference in the association between insomnia symptoms and mental health was not substantial, males had slightly higher odds of anxiety and depression associated with insomnia symptoms than females. A female’s lifespan is marked by experiences and challenges beginning from adolescence, biological changes, gender roles, hormonal changes, childbirth and child rearing, and menopause, with all carrying potential risks of insomnia. As such, females may cope better with insomnia than males, as they may experience insomnia at an earlier age than males or throughout their lifespan, and may develop better resilience or mental health-seeking behavior [21]. In addition, studies support that males self-report mental health and insomnia issues less frequently than females [22]. Moreover, males may experience greater societal pressure and expectations, which could limit opportunities to develop social connections [23]. Social support and networks have been shown to contribute positively to mental health and well-being [24]. Our finding underscores the importance of more awareness around insomnia and mental health, regardless of sex.

Age differences in the association between insomnia symptoms and mental health were substantial, indicating higher odds for younger adults compared to older adults in both males and females. This finding, coupled with the lower mean age in those with insomnia symptoms, suggests that the burden of insomnia is not only higher in younger adults but also more linked to poor mental health. This is particularly alarming given that younger adults make up the core of the workforce essential to maintaining and advancing societal needs. Efforts to reduce stress and burnout and improve work–life balance would likely contribute to a better quality of life and well-being.

In those with insomnia symptoms, females were more likely to receive mental health care, but also more likely to delay mental health care due to financial barriers than males. The reasons that females exhibit higher levels of mental health-seeking behavior compared to their male counterparts may rely on their attentiveness to their internal psychological experiences compared to males. Societal and cultural expectations of hypermasculinity may prevent males from expressing their emotions and seeking help, especially about mental health, as they risk being perceived as weak. Sigma related to mental health, shame, doubts about the effectiveness of mental health care, costs, and other barriers are still prominent [25]. Reducing those barriers and improving mental health care access is paramount to a better society, given the high prevalence of mental health disorders.

Females were about two times more likely to experience financial barriers to mental health care than males. In the U.S., females may have lower financial earnings and resources compared to males, which could play a critical role in limiting access to health care [26]. Reducing income gap disparities will likely contribute to improving access to mental health care. Alternatively, females may encounter more barriers, possibly because their greater awareness of mental health issues and higher likelihood of seeking care make such challenges more apparent compared to males.

We further observed that the female–male differences in mental health care utilization and perceived barriers were only minimally explained by health insurance coverage and area of residence. Additional barriers such as stigma, cultural attitudes, negative perception of treatment, minimization of insomnia symptoms, provider accessibility, or availability could not be assessed in the present study and should be considered in future research.

Our findings further underscore the benefits of making sure insomnia assessments are not overlooked, with diagnoses slipping through the cracks leading to significantly negative health outcomes. Beyond mental health, insomnia has been linked to multiple other chronic diseases, such as cancer, stroke, heart failure, and hypertension [27,28,29,30]. The benefits of interrupting insomnia symptoms early in the disease progression cannot be understated, whether by prevention or intervention. By making an appropriate diagnosis of insomnia, there is a chance of targeted clinical intervention, including cognitive behavioral therapy, instead of hoping that insomnia will resolve spontaneously.

Several limitations should be considered when interpreting these findings. This was a cross-sectional study, which limits our ability to determine directionality and establish causal inference. Insomnia symptoms and mental health outcomes were assessed concurrently, and it is unclear which preceded the other. Future studies with longitudinal data are needed to confirm those findings. Furthermore, the survey was based on self-reported data, which are subject to misclassification bias of both the exposure and the outcome. Future studies and data collection should consider including more objective measurements of sleep and mental health. Insomnia symptoms were compiled into a symptom index, which assumed that all symptoms have equal health implications. An analysis by type of insomnia symptom indicated higher odds for trouble initiating sleep compared to trouble maintaining sleep and non-restorative sleep. However, all three individual insomnia symptoms included in the composite score were strongly associated with anxiety and depression. In addition, insomnia symptoms, rather than diagnosed insomnia, were assessed. This research is an observational study and should be interpreted in light of the potential for residual or unmeasured confounding that may influence the observed associations. Lastly, this study focuses solely on the U.S. adult population, and its findings may not be generalizable to other populations with different health care systems and cultural contexts.

Despite these limitations, the NHIS is a large sample, and the design allows for making inferences about the U.S. adult population. This study was innovative in highlighting age and sex differences in the association between insomnia symptoms and further shedding light on mental health care utilization and barriers. These findings have significant implications for public health, policy, health care, and future research. Public health interventions could include raising awareness about insomnia symptoms and mental health, and improving access to mental health care. Policies aimed at improving access to mental health care could include expanding insurance coverage to encompass cognitive behavioral therapy, as well as extending overall health insurance coverage. Mental health counseling and therapy involve diverse professionals, such as psychiatrists, psychologists, primary care providers, nurses, and clinical social workers, who could all benefit from interprofessional collaboration. In rural areas, for example, where specialist availability is scarce, it often rests on the primary care provider to provide a range of care, including mental health care.

## 5. Conclusions

The association between insomnia symptoms and increased odds of anxiety and depression in both females and males suggests that insomnia symptoms may be an indicator of mental health outcomes regardless of sex. Insomnia symptoms are more strongly associated with mental health in younger adults compared to older adults. Public health interventions should consider targeting younger adults. The study also revealed that females with insomnia symptoms have higher odds of receiving mental health care, but are also more likely to delay treatment due to costs compared to their male counterparts. This underscores the importance of addressing the gender financial gap to ensure affordable mental health care options for females and improving mental health-seeking behaviors for males. Holistic approaches, including prevention, destigmatization, identification, normalizing help seeking, and improving access to mental health care, are warranted.

## Figures and Tables

**Table 1:** Baseline characteristics by insomnia symptom scores.

	Insomnia Symptom Scores			
Characteristics	0 n (Weighted%) ^a^: 2944 (11.1)	1–4 n (Weighted%) ^a^: 18,898 (71.1)	5–9 n (Weighted%) ^a^: 4849 (17.8)	Total n (Weighted%) ^a^: 26,691 (100)
**Age** (in Years)				
Mean (95% CI)	51.6 (50.6, 52.5)	47.7 (47.3, 48.0)	48.4 (47.8, 49.1)	48.2 (47.9, 48.5)
**Sex**				
Female	1389 (42.8)	10,110 (50.2)	3052 (61.4)	14,551 (51.4)
Male	1554 (57.2)	8787 (49.8)	1796 (38.6)	12,137 (48.6)
**Race and ethnicity**				
Hispanic	626 (26.6)	2575 (13.3)	589 (14.5)	3790 (17.1)
Non-Hispanic Asian	297 (10.5)	1161 (6.1)	150 (3.0)	1608 (6.0)
Non-Hispanic Black	409 (13.9)	2056 (11.6)	452 (10.2)	2917 (11.6)
Non-Hispanic Other ^b^	71 (2.8)	433 (2.5)	158 (3.8)	662 (2.8)
Non-Hispanic White	1541 (46.2)	12,673 (63.4)	3500 (68.8)	17,714 (62.5)
**Education**				
Less than high school	420 (18.2)	1376 (9.4)	446 (10.8)	2242 (10.6)
HS graduate/GED	880 (31.7)	4502 (25.7)	1308 (29.4)	6690 (27.0)
Some college	395 (14.3)	2713 (15.9)	855 (19.0)	3963 (16.3)
Associate	343 (11.7)	2500 (13.3)	679 (14.0)	3522 (13.2)
Bachelor	530 (15.1)	4641 (22.0)	949 (17.1)	6120 (20.3)
Graduate degree	356 (9.1)	3084 (13.8)	583 (9.7)	4023 (12.6)
**Ratio family income to poverty threshold** ^c^				
<1 (below poverty level)	368 (12.4)	1582 (8.3)	692 (12.7)	2780 (9.5)
1	604 (21.7)	3055 (16.4)	993 (20.4)	4839 (17.7)
2	488 (117.3)	2793 (15.1)	872 (19.5)	4313 (16.1)
3	403 (114.1)	2538 (12.9)	609 (12.7)	3680 (13.0)
4	292 (9.4)	2221 (12.0)	469 (9.8)	3071 (11.3)
≥5	789 (25.0)	6709 (35.3)	1214 (24.9)	8968 (32.3)
**Marital status**				
Married	1407 (53.6)	8918 (52.7)	1920 (45.9)	12,245 (51.6)
In couple	144 (6.8)	1243 (8.8)	371 (10.5)	1758 (8.9)
Neither	1346 (39.6)	8528 (38.4)	2503 (43.6)	12,377 (39.5)
**Smoking status**				
Current	314 (10.7)	1924 (10.1)	855 (17.6)	3093 (11.5)
Former	698 (21.4)	4563 (21.4)	1420 (27.0)	6681 (22.4)
Never	1928 (67.8)	12,378 (68.5)	2566 (55.4)	16,872 (66.1)
**Alcohol consumption**				
Abstainer	652 (24.5)	2102 (12.9)	385 (8.8)	3139 (13.4)
Former	643 (20.5)	3327 (15.7)	1115 (21.1)	5085 (17.2)
Current	1637 (55.0)	13,386 (71.4)	3332 (70.1)	18,355 (69.4)
**Physical activity**				
Inactive	859 (30.2)	4657 (24.4)	1781 (36.2)	7297 (27.2)
Insufficiently active	654 (21.9)	4939 (26.1)	1237 (25.6)	6830 (25.5)
Sufficiently active	1385 (47.9)	9120 (49.5)	1791 (38.2)	12,296 (47.3)
**BMI Group**				
Underweight (BMI ≤ 18.4)	46 (1.6)	295 (1.7)	72 (1.8)	413 (1.7)
Healthy weight (18.5–24.9)	1001 (35.0)	5961 (32.0)	1254 (26.5)	8216 (31.3)
Overweight (25–29.9)	1072 (37.1)	6375 (34.0)	1521 (30.9)	8968 (33.8)
Obese (BMI ≥ 30)	745 (26.4)	5894 (32.4)	1892 (40.8)	8531 (33.2)
**Sleeping pills**				
Never	2749 (94.3)	15,599 (84.0)	2820 (60.0)	21,218 (80.9)
Some days	48 (1.6)	1817 (9.1)	777 (15.8)	2646 (9.5)
Most days	13 (0.4)	311 (1.5)	278 (5.7)	602 (2.1)
Every day	134 (3.6)	1169 (5.4)	970 (18.5)	2280 (7.5)
**Diabetes**				
Yes	278 (8.5)	1842 (8.7)	716 (13.5)	2836 (9.6)
**Hypertension**				
Yes	1019 (30.0)	6673 (30.5)	2113 (39.4)	9805 (32.0)
**Elevated cholesterol**				
Yes	810 (24.0)	5808 (26.4)	1832 (33.3)	8450 (27.4)
**Heart disease** ^d^				
Yes	207 (5.6)	1381 (5.8)	569 (10.0)	2157 (6.5)
**Stroke**				
Yes	123 (3.5)	581 (2.3)	255 (4.3)	959 (2.8)
**Cancer**				
Yes	304 (7.8)	2294 (9.1)	738 (12.8)	3336 (9.6)

^a^ weighted to account for complex sampling design; ^b^ other single and multiple races; ^c^ ratio of family income to poverty threshold for sample adult’s family; ^d^ coronary heart disease, angina, heart attack. Data Source: National Center for Health Statistics, National Health Interview Survey, 2022.

**Table 2:** Association between insomnia symptom scores and mental health (1 unit increase in insomnia score).

	Crude	Adjusted
Females		
Anxiety		
All	1.39 (1.36, 1.43)	1.28 (1.24, 1.31)
Age < 50	1.45 (1.40, 1.50)	1.34 (1.29, 1.40)
Age ≥ 50	1.35 (1.31, 1.39)	1.20 (1.16, 1.24)
Depression		
All	1.44 (1.41, 1.47)	1.31 (1.28, 1.35)
Age < 50	1.51 (1.46, 1.57)	1.40 (1.35, 1.46)
Age ≥ 50	1.37 (1.33, 1.41)	1.22 (1.18, 1.26)
Males		
Anxiety		
All	1.42 (1.38, 1.46)	1.30 (1.26, 1.34)
Age < 50	1.48 (1.42, 1.55)	1.37 (1.30, 1.43)
Age ≥ 50	1.36 (1.31, 1.41)	1.22 (1.17, 1.28)
Depression		
All	1.46 (1.41, 1.50)	1.33 (1.28, 1.37)
Age < 50	1.51 (1.44, 1.57)	1.37 (1.31, 1.44)
Age ≥ 50	1.41 (1.35, 1.47)	1.27 (1.21, 1.33)

Adjusted for age, race and ethnicity, education level, marital status, ratio of family income to poverty threshold, smoking, alcohol consumption, physical activity, body mass index, diabetes, hypertension, heart disease, stroke, cancer, and sleeping pill utilization. Data Source: National Center for Health Statistics, National Health Interview Survey, 2022.

**Table 3:** Mental health care utilization and perceived barriers in females vs. males among those with insomnia symptoms.

	Crude	Adjusted
Received mental health care in the past year	1.58 (1.43, 1.73)	1.70 (1.53, 1.87)
Delayed mental health care because of cost	1.85 (1.60, 2.15)	1.96 (1.67, 2.30)
Needed mental health care but did not get it because of the cost	1.97 (1.70, 2.28)	2.14 (1.82, 2.50)

Adjusted for age, race and ethnicity, education level, marital status, ratio of family income to poverty threshold, smoking, alcohol consumption, physical activity, body mass index, diabetes, hypertension, heart disease, stroke, and cancer. Data Source: National Center for Health Statistics, National Health Interview Survey, 2022.

**Table 4:** Mediation and moderation of mental health care utilization and perceived barriers.

	Natural Indirect Effect (OR, 95% CI)	% Mediated (%, 95% CI)	*p*-Value Interaction
Received mental health care in the past year			
Insurance coverage	1.01 (1.01, 1.02)	2.08 (1.40, 3.02)	0.3145
Residence (metropolitan and nonmetropolitan)	1.00 (1.00, 1.01)	0.72 (0.12, 1.55)	0.3700
Delayed mental health care because of its cost			
Insurance coverage	0.98 (0.97, 0.99)	−3.70 (−6.42, −2.08)	0.4278
Residence (metropolitan and nonmetropolitan)	1.00 (1.00, 1.01)	0.42 (0.04, 1.16)	0.3891
Needed mental health care but did not get it because of the cost			
Insurance coverage	0.98 (0.97, 0.99)	−3.17 (−5.23, −1.83)	0.5826
Residence (metropolitan and nonmetropolitan)	1.00 (1.00, 1.01)	0.49 (0.04, 1.14)	0.8464

Adjusted for age, race and ethnicity, education level, marital status, ratio of family income to poverty threshold, smoking, alcohol consumption, physical activity, body mass index, diabetes, hypertension, heart disease, stroke, and cancer. Data Source: National Center for Health Statistics, National Health Interview Survey, 2022.

## Data Availability

The data used for this analysis are publicly available at https://www.cdc.gov/nchs/nhis/index.html (accessed on 8 January 2025).
